# Correction: Using systems-mapping to address Adverse Childhood Experiences (ACEs) and trauma: A qualitative study of stakeholder experiences

**DOI:** 10.1371/journal.pone.0315261

**Published:** 2024-12-05

**Authors:** Thi Hoang Vu, Jared Bishop, Leigh McGill, Luke Valmadrid, Shelley Golden, Dane Emmerling, Seth Saeugling, Vichi Jagannathan

Vichi Jagannathan is not included in the author byline. Vichi Jagannathan should be listed as the eighth author and affiliated with MBA, Co-CEO of Rural Opportunity Institute and Adjunct Professor at the UNC-CH Gillings School of Public Health. The contributions of this author are as follows: Formulated overarching research goals and aims, acquired financial support, developed methodology, managed and coordinated responsibility for the research activity planning and execution, provided study materials, oversaw the research planning and execution, verified the overall replication/reproducibility of results/experiments, prepared data presentation and wrote the critical review and revision.
