# Supplementary material for: Using systems-mapping to address Adverse Childhood Experiences (ACEs) and trauma: A qualitative study of stakeholder experiences
Source: PLoS One. 2022 Aug 18;17(8):e0273361. doi: 10.1371/journal.pone.0273361 (PMC9387783; doi:10.1371/journal.pone.0273361)
Supplement: S1 Table — (DOCX) [file pone.0273361.s001.docx]

|  | All participants who were engaged in the systems-mapping project (N = 413) | Participants who were engaged in the systems-mapping project interviewed (N = 8) |
| --- | --- | --- |
|  | N (%) | N (%) |
| Sector |  |  |
| K-12 Schools | 96 (23.3%) | 1 (12.5%) |
| Non-Profit | 83 (20.2%) | 2 (25%) |
| Health | 57 (13.7%) | 1 (12.5%) |
| Early Childhood Education | 37 (8.9%) | - |
| Social Services | 35 (8.5%) | 1 (12.5%) |
| Elected Officials/Government | 19 (4.6%) | 1 (12.5%) |
| Local Businesses | 17 (4.1%) | - |
| Higher Education | 16 (3.9% | 1 (12.5%) |
| Juvenile Justice | 15 (3.6%) | - |
| Substance Misuse Treatment | 14 (3.5%) | - |
| Faith | 9 (2.1%) | - |
| Law enforcement | 7 (1.8%) | 1 (12.5%) |
| Transition Re-Entry | 7 (1.8%) | - |
| Race |  |  |
| Black | 240 (58.1%) | 5 (62.5%) |
| White | 100 (24.2%) | 3 (37.5%) |
| Multiple races | 38 (9.3%) | - |
| Hispanic | 15 (3.7%) | - |
| Native American | 10 (2.5%) | - |
| Asian | 10 (2.5%) | - |
| Gender |  |  |
| Female | 279 (67.6%) | 1 (12.5%) |
| Male | 132 (31.9%) | 7 (87.5%) |
| Non-binary | 2 (0.5%) | - |
