# Supplementary material for: Using systems-mapping to address Adverse Childhood Experiences (ACEs) and trauma: A qualitative study of stakeholder experiences
Source: PLoS One. 2022 Aug 18;17(8):e0273361. doi: 10.1371/journal.pone.0273361 (PMC9387783; doi:10.1371/journal.pone.0273361)
Supplement: S2 Table — (DOCX) [file pone.0273361.s002.docx]

**Interview guide for participants who directly participated in the systems-mapping project**

| Abbreviation | |
| --- | --- |
| RQ | Research question |
| IQ | Interview question |

**Introduction**

Good Morning/Afternoon! My name is [interviewer’s name]. I am a student at [redacted[] helping the Rural Opportunity Institute (ROI) understand how participants of the systems mapping process in [county name redacted] were impacted by the systems mapping process. By “systems mapping process” we mean the series of meetings that [ROI] hosted in 2017 and 2018 to hear people’s stories, do activities, and build a visual map to identify the forces that were causing ACEs, trauma, and healing, in people’s lives.”

We would also like to ask you if you would be comfortable with us recording the audio and video of our conversation today, so that we can make sure we reflect your thoughts and opinions accurately. All the information I receive from you, including the audio recording, will be strictly confidential and kept on a password protected, secure worksite. All names mentioned will be removed from the transcription of the interview, and the audio recordings will be destroyed after transcription.

Obtain verbal consent.

We really appreciate having you today. We acknowledge that the systems mapping process took place a few years ago. There are no right or wrong answers during this conversation, and we encourage you to only share what feels comfortable. That’s all we have for the introduction; do you have any questions for us before we begin? (If no questions, let participants know that you will begin recording now.)

**RQ1. Pre-Process Questions**

IQ1-1. How long have you lived in the area?

IQ1-2. What’s your favorite part of living here?

IQ1-3. How did you get introduced to the systems mapping process, the Rural Opportunity Institute, or [Rural Opportunity Institute organizers]?

IQ1-4. How were you involved in the systems mapping process?

IQ1-5. What motivated you to agree to participate in the systems mapping process? What did you hope to change or gain by participating?

*Probes*: Did you feel a connection between the systems mapping process and your life or work?

**RQ2. Process Questions**

**(If over Zoom, provide picture of the systems map)**

IQ2-1. What was your favorite part or parts about the process?

IQ2-2. What did you not enjoy or find difficult? What could have been better?

IQ2-3. Could you describe a challenge in the systems mapping process? What do you think was a weakness of the project?

*Probe:* How were these challenges/weaknesses addressed, or were they not addressed?

IQ2-4. What parts of the systems mapping process did you feel like were the most relevant to you and why?

IQ2-5. What did it feel like to participate in the system mapping process? What were the positive emotions / what were the negative emotions?

*Probe:* If so, from what, from whom?

IQ2-6. Were there any times during the systems mapping process when there were information or people you felt should be included, but were not included?

*Probe:* If so, could you describe that?

IQ2-7. How if at all did you feel like ROI leadership were open to hearing and then incorporating your feedback during the mapping process?

*Probe*: What perspectives might have needed more time, if any?

IQ2-8 What parts of the systems mapping process did you feel like were the most relevant to addressing trauma and why?

IQ2-9. What parts of who you are did you share throughout the process?

*Probe*: Could you describe a specific moment that you recall sharing a personal experience or aspect of your personal identity?

*Probe*: How did you see a part or parts of your identity included in or changing the systems mapping process, or the systems map itself?

**Transition:** Now I’d like to ask you about how you feel the systems mapping process, and your participation in it, changed you and your community.

**RQ3. Impact and Outcomes Questions**

IQ3-1. [Recalling the question about what they thought they could change or gain by participating], did participating in the systems mapping process fulfill your goals for participation?

IQ3-2What personal value, if any, was created for you by participating in this process?

IQ3-3What community benefit, if any, have you seen come as a result of the systems mapping process?

*Probe*: What new value, if any, was created in the community outside of the ROI space, and how did being a part of the process impact others, their work, or their actions?

*Probe*: What was your biggest take away? What, if anything, did you learn from this process?

IQ3-4. If anything, what is different in your life or the lives of people you know as a result of being part of the systems mapping process?

*Probe:* With whom, if anyone, have you discussed the process, outside of the participating group? Could you describe that discussion?

*Probe:* Since participating in the systems mapping process, did you feel less, more, or the same in the level that you felt connected to the [county name redacted]?

**RQ4. Questions about Recommendations**

IQ4-1. Compared to other community health efforts you have witnessed or experienced, what was different about ROI’s approach?

IQ4-2. Would you recommend the systems mapping process you experienced to other communities that want to improve community health? If so, why?

*Probe:* Potential follow-up: How do you think the process might be helpful to others that try it?

IQ4-3. Do you have any advice for other communities if they did their own systems mapping process?

**Conclusion**

- Is there anything that you would like to share that we did not discuss?
- Do you have any other questions for us?
- Thank you for taking the time to talk to us today!
- Gift card notification.
- We greatly appreciate your time.

**Demographic Questions**

- Age
- Highest level of education
- Race
- Gender you identify with

**Interview guide for participants who were involved in initiatives informed by the systems-mapping project**

**Introduction**

Good Morning/Afternoon! My name is [interviewer’s name]. I’m part of a group of students at [redacted] helping the Rural Opportunity Institute (ROI) understand how ROI stakeholders in [county name redacted] were/are impacted by the systems mapping process. By “systems mapping process” we mean the series of meetings that [ROI] hosted in 2017 and 2018 to hear people’s stories, do activities, and build a visual map to identify the forces that were causing ACEs, trauma, and healing, in people’s lives.”

We would also like to ask you if you would be comfortable with us recording the audio and video of our conversation today, so that we can make sure we reflect your thoughts and opinions accurately. All the information I receive from you, including the audio recording, will be strictly confidential and kept on a password protected, secure worksite. All names mentioned will be removed from the transcription of the interview, and the audio recordings will be destroyed after transcription.

Obtain verbal consent.

We really appreciate having you today. We know that you were not directly involved in the systems mapping process that took place a few years ago, but we are very interested to learn what your experience has been like working with ROI. There are no right or wrong answers during this conversation. That’s all we have for the introduction, do you have any questions for us before we begin?

All right, I am going to start the recording now, is that all right with you?

**Pre-Process Questions**

- How long have you lived in the area?
- What’s your favorite part of living here?
- How did you get introduced to ROI’s work?
- What has motivated you to work with ROI?

**Past Work Questions**

- Could you describe the work you have done with ROI?
- What have been the highlights for you of working with ROI?
- What do you enjoy most about working with ROI?
- What do you enjoy least?
  - What do you wish was different about how ROI works?
- How would you describe ROI’s work to someone who has never heard about it?
- How would you describe trauma and resilience to someone who is not familiar?

**Strategy / Process Questions**

**(If over Zoom, provide picture of the systems map)**

- Could you describe the strategy that ROI is following around trauma & resilience?
  - Who built the strategy?
- How is the strategy connected to, or not connected to, the work you do with ROI?
- Did you know ROI did a systems-mapping process from 2017-2018?
  - If yes, ask “what do you know about that process?”
  - If no, display systems-map and provide brief overview of work that has been informed by the mapping project.
  - What was the impact of doing a systems-mapping process?
- What parts of yourself (parts of your identity, who you are), or any past experiences are you able to bring to your work with ROI?

**Impact and Outcomes Questions**

- What value was created for you by participating in this work around trauma and resilience?
- If ROI had not done the mapping process, what might be different in your current work?
  - If any, did you notice a difference in the level of connection between yourself or others, to the work because of the systems mapping process?
- What benefit, if any, have you seen come as being a part of this work around trauma and resilience?
  - Talking about this work around trauma and resilience, what do you believe is its’ greatest contribution to [county name redacted]?
  - How has your knowledge changed, by participating in this work?
- If anything, what is different in your life or the lives of people you know as a result of being part of this work?
  - Since participating in this work, did you feel less, more, or the same in the level that you felt connected to the [redacted] community?

**Questions about Recommendations**

- Compared to other community health efforts you have witnessed or experienced, what is different about ROI’s approach, this work around trauma and resilience?
- How do you think this work around trauma & resilience could be helpful to other communities?
- Do you have any advice for other communities going about their own work around trauma & resilience?

**Conclusion**

- Is there anything that you would like to share that we did not discuss?
- Do you have any other questions for us?
- Thank you for taking the time to talk to us today!
- Gift card notification
- We greatly appreciate your time.

**Demographic Questions**

- Age
- Highest level of education
- Race
- Gender you identify with
