# Supplementary material for: Using systems-mapping to address Adverse Childhood Experiences (ACEs) and trauma: A qualitative study of stakeholder experiences
Source: PLoS One. 2022 Aug 18;17(8):e0273361. doi: 10.1371/journal.pone.0273361 (PMC9387783; doi:10.1371/journal.pone.0273361)
Supplement: S3 Table — (DOCX) [file pone.0273361.s003.docx]

| **Theme** | **Quotes** |
| --- | --- |
| **Aim 1:** Impact of systems-mapping project | |
| Recognition and understanding of own trauma | The thing that probably affected me personally was a lot of these things I know from my background personally and some of those things I had to deal with, you know, even as a older adult, you know, you still deal with these traumas that may be buried, you know; and so, you know, you know, a lot of things came back and having to deal with those things or rethink, you know, and so that was probably the most difficult. (Higher education representative)  I was part of a group, we went to Tarboro. And met over there and [ROI] had these maps, all you know PowerPoints all around. And I looked at ‘em and I could see people almost looking cross-eyed and I sat back and I thought, ‘I understand this.’ I understand it, and it all makes, all makes sense to me. It’s tragic. But I understand why; how the anger gets out, how the hopelessness and why and what you have to do, which is, which was, I'm not going to say it's 180 degrees, different from my former thinking, but it was probably at least 120 degrees different. And just listening to it...it just, it resonated with me. And it made, it made sense. And that day is when I, when I really understood that it did because...I thought ‘I got this. I got this’. (Local government employee #1)  I think that we need to make sure that people understand that it can happen to everyone, that, um, it's not somebody else's problem. It's the community's problem. And lots of people have experienced trauma, but they've kept it to themselves. And they, I mean, that's proven on the ACEs score, that a lot of people have experienced trauma and just never discussed it...but just help people understand that there's nothing to be ashamed of, that what they've experienced is not their fault. Um, yeah, kind of removing the blame. (Community college staff member)  I think getting different perspectives on what they thought trauma was and then being educated on what trauma is and a lot of people in the room...Something would be diagnosed as trauma, growing up, it was just a way of life. I mean it, this is what it is. So you don't see it being something that's trauma or whatever because your friends are going through the same thing you were going through. So this is like a way of life versus, versus, you know, something that shouldn't have to take place, you know? And I think that process kind of opened some eyes in the room on what adverse childhood trauma is. (Nonprofit employee)  Well, the personal value for me was that it gave me an opportunity to really look at myself to look at the weaknesses that I had that I didn't address and also it made me look at the strength that I had that I can use to overcome those weaknesses and, and that's that's what it did, it made me basically an open book I look first at myself. (Community volunteer)  Well, I really got to go inside my, my communities’ homes, but not just their homes but their mindset and or how they set up a home because of the systemic things set in place, but that was the downside about that broke my heart. Because even though they seem good on the outside my people are still crying on the inside. (Youth mentor) |
| Connection to other community members | I feel like I'm part of that system. I feel more like I'm a part of the community. I feel More related to other members of the community, even if I can't remember all the names or even the faces necessarily I can remember what we did together. There's a bit of a natural alliance, as I encounter people going forward in the future. Oh, yes, we work together on the ROI project...There was a very diverse group everybody had a little piece of knowledge to add to that systems map. And people getting to know each other who didn't know one another before… So community bonding, I would say...,maybe that'll enable us to affect social change more effectively. (Physician)  So there are things, for example, like relationships built for community members that would have never spoken before, so like I as a principal wouldn't necessarily have navigated. But now all of a sudden I have these connections. So we're able to build partnerships for things I didn't know existed, you know. (Principal)  I got to know some people better than I ever thought even [name redacted], who was, we were good friends before I mean, you know, we're, you know, we talk all the time about stuff that we never talked about and and so it, it allowed it allowed people to to come together to share, but also gave them an opportunity to realize, too, that we're not so different, you know, and, and so we have, I believe it's given the community a better opportunity to become more intimate with each other and understand and understand that if I hurt, everybody's hurting you know so and if I can't figure it out, then we're all lost. (Higher education representative)  I think it was a good reminder to keep an open mind with people. I try to do that anyway, but I think this was a very good reminder that you know everybody has their own stuff that they're dealing with and being kind can't hurt. (Local government employee #2)  Okay, and the good part about is that, you know, I got to be more empathetic. The good part is, like I said, I got to be more empathic, I got to understand it. Even if my neighbor has an issue, my issue is not with my neighbor of mine, my issue is with the people that put the systems in place because my neighbor is a criminal, because I have stuff and if my neighbor was envious of what I have, it’s not my fault. That was the point, but just to see that. My fight is not my people. (Youth mentor) |
| **Aim 2:** Leveraging systems thinking to address ACEs and trauma | |
| The role of organizers | Oh, they were very, they were very open to feedback and because with the feedback they could improve on what they would do if we gave them honest feedback and the leadership were always open to all, sometimes even criticism about the program. And they, they always willing to fix whatever they thought might be broken. So they are very, very open to, you know, make an adjustment to, you know, to help the most people. (Community volunteer)  Well, [organizers] were very interested in what I had to say. Yeah, I thought they were open to comment. And I didn't feel like they were resistant to anything I had to offer. (Physician)  I think that they also took risks by making themselves really available in the community. So it wasn't just like they got they learned about it. It was like you saw them at like community events and they became like active interested participants of the community they serve. I didn't feel like I was like under a specimen. You know, it felt like they joined us in the work. And I think that's, that's really important. Yeah, you know, now that I'm saying that out loud what I think that created was just like permission to be vulnerable because it never felt like we were being judged. It felt like the questions they were asking really came from the curiosity to understand the historical context. (Principal)  They met with everybody you know and you know you meet with people you; Let them know you're, you're not here to tear down, you're here to build them up, you're here to participate. Not, you know, observe and You want to pull people together not, you know, pull them apart, you want to, you want to be a part of the solution, not a part of the problem you, you want to help, not hurt. You know, so all those things. I think they did that and they took their time and they listen, listen to everybody. (Higher education representative) |
| Diverse voices and representation at the table | I really like the affirmation of one, just who they invited to the table. That was the first time that I really internalized the fact that I wasn't alone in this work. And it was very interesting to hear from healthcare professionals or probation officers, or clergymen and to be talking about the same area and the same issues, but I thought through the lens of not only how they impact events, but what they were doing about it. And so it was the first time that I saw that like the issue that I see can be solved in more than one way. And I think that that gave me a lot of hope. So unlike the surface level. I think it was like very engaging to know that this was a community doing community work. (Principal)  I found it very uplifting. It was, I enjoyed working with a diverse group of people. I enjoyed being a primary care physician. I have met a lot of people over time in the community and it's good to see some of those same people over time in different settings and to be able to work together with them. It was rewarding for me to be able to work with [ROI] it was, as I already said it very rewarding to me to be exposed to a new idea totally novel to me really a new way of getting at the truth. (Physician)  I'm a people person, so I like to bring people together. You know, and they were bringing people together who I normally don't work with a, you know, to come to the table to come up with some solutions to help you know better than the community. (Nonprofit employee) |
| Gaps in community representation | Well, I think the system makers, the board makers, the people that's on the board and all that, you know, put the systems in order and then none of them was at the table. We're always talking to the people that was affected by the system, but nobody that was no serious players in place ever came to the table here. (Youth mentor)  The children were missing. Hmm, we would talk because we were talking, we were talking about. We were talking about the children and a lot of problems and issues that the children had but eventually the children were born into it because the program was set up in the school to deal with the trauma. That they have But other than other than that, again, I still say the parents in general were not really included as they should have. (Community volunteer)  Um, I think the marginalized, those folks who are marginalized don't always have an opportunity to express themselves. (Higher education representative)  I think in this day and time, with some of the larger companies like around here: Cummings, Pfizer, Honeywell, the hospital, Nash, UNC, and some others. I think at the lowest point, their HR person at the lowest. And it would be great to have their senior management, one or two from senior management there as well. I think, um, some retired educators that have the time and the skillset and they have the passion for it. They retired from teaching, but like my late mother was a teacher 40 years, that can maybe give some guidance. (Local government employee #1)  Yeah, like I said, I think that probably could have had a little bit more influence from Rocky Mountain. Because of where it was located...because you have you can do something in Edgecombe County and it's going to affect Nash County, too, because they're just twin County. (Nonprofit employee) |
| Talking about trauma can itself be traumatic | I'm remembering some of our conversations...a lot of is individualized and the emotions that I had to deal with were, I'm happy that I was able to have been able to cope with a lot of these before our conversation... but, but even now, I still you know, are dealing with some of those things emotionally trying to work through some of those things. You know, this year I lost my mother, I lost my brother, and, and it kind of made you revisit some of those things. (Higher education representative)  I think just inherently when you spend time discussing the realities of the world, it can become overwhelming. And so I remember there was pieces of like going through the process to really see how things that we do or systems that are created in a community like perpetuate negative things. And so when you think specifically about like adverse childhood experiences and the trauma, there's like compounding factors. And I found that the avenues in which I served in terms of like education and what we were doing, we were a large part of that through things like suspensions. And lack of community engagement and lack of social awareness and I think we just like contributed to that in a lot of ways. In terms of like, you know, with the best of intentions but you saw it show up in a way that just kind of was overwhelming to know that you're like in a system that's perpetuating this. (Principal)  Yeah, so it had me thinking a lot, you know...during that time, you know, and I think I was having a lot of flashbacks and I remember some of the things that I went through as a child--how it made me feel, what was some of the effects of it, you know, how it had an effect on my life today. (Nonprofit employee) |
| Visualizing systemic sources of trauma | Um, if you look at the map itself. Are these areas that have like plus sign and minus sign. And  that's kind of like, you know, the areas that the loop is reinforced or weakened and I thought  that it was just really interesting to think about. It's just life right that like right there are some  things compounded that are good and some things that are compounded that are bad. And let's  make meaning of the outcome. And I just thought that was really interesting reflection, because  again I hadn't considered that before...This is like super interesting to see all those circles and  cycles working in tandem to create the site problem and or solution based on how you look at it.  Yeah, so I just really like the idea of creating a map of how these things are all interrelated and  then again like getting curious about, like, then what can you, what can you are aware, maybe  it's an appropriate Thought to, like, where do you intervene, then in this and I never thought  about that before. (Principal)  The thing that helped me the most was the visual. When, when they took this information and  processed it and  put it on paper, not just sticky notes, but putting in a diagram. And we talked  about each segment of this diagram and it got more and more and more. But yet, is it got  complicated I can actually see it better...So the visual for me was probably the most impressive  and and their ability to you know, just put it in a situation where, you know, it wasn't a thought  on the way home, and I write on a note paper and and lose the note paper, but they were able to  keep everything and and document it and put it in a situation where, you know, where we could  see and evaluate it and reevaluate it. (Higher education representative)  I think we sat down at tables of about four people each. And somehow we talked a bit about  who we were and what our disciplines. We had lots of little pieces of paper. We started writing  down ideas. We presented our ideas to the group and then we started adding them to this huge  paper chart. That showed what the issues and what the forces were in the community that were r  related to toxic stress. (Physician)  I don't think I would have a framework to address. I think it would still be putting out fires, to use an analogy, instead of a process that has the beginning and you make progress as you work through it. Like you may not, you know, solve the problem, but we have, we have a framework within which to work and to make continual progress with. Even though it's gradual, you can see the progress, you can see the benefit, even though it doesn't solve a problem, you know, and I think maybe without ROI, I would still be trying to solve problems with one little quick fix. (School guidance counselor #1) |
| Incorporating SMP skills into everyday and professional life | Cause the school system has come aboard now and [ROI has] introduced the biofeedback program to schools, and a lot of schools and principals have come aboard and really want to implement these practices and tools to their students, so it’s a really good impact. (Pastor)  We want students to understand that we know that you go through things as well. We, we know that you have things that you're dealing with. But when you come to school, ‘how can we make school life better, even though you have these things going on?’ And building that relationship with, with the students in order to allow them to be the best student as a whole, instead of just their academics. ‘How is home?’ You know, ‘is there anything that I can do to help you to help make things better for you at home,’ as opposed to just come into school learning math, science, social studies and language arts. (School guidance counselor #1)  It's given me confidence in working with clients...I'm learning new things and...I would say it's given me some renewed confidence and working with children and adults that have been impacted by trauma. (Health Care Provider)  I think that the impact on that small group of people is big, because I think that it, it’s information which empowers people…I'm more mostly changing the mindset of people so that they understand that to get students to learn the most, which is our goal, you have to put them in an environment that they're comfortable and that they feel safe and they feel the most safe when they know that the response of a teacher, even when they make a mistake that the response of the teacher is going to be a safe response…And I just think that kids need to be aware. We have a lot of children that it is part of their lifestyle to get angry and upset.And The first step is for them to be aware that that's a negative behavior and that there is another way to do that. There is another way to react, even though you have the same feelings, but your reaction can be different. (Teacher)  I don't know just connecting, connecting, it sounds crazy, but just connecting systems like mapping out how can we all support and benefit each other. (School guidance counselor #1)  You know, I think, in doing that mapping process, [ROI] integrated key players. It allowed them and help them to buy into the whole idea, to the whole process. So it became a way of uniting all the key players in this process...So, you know, people start gaining interest and they start gaining having a voice and by having that voice, they have ownership to what's being done with being said, which is going to increase their commitment. So I don't think I don't think they would have the, the commitment, the level of buying had they not done that mapping process. (Counselor and licensed therapist)  … there are many stakeholders in education. We have families. We have teachers. We have community members, social workers, nurses, people that all play a part in a child's experience in the school setting. And as we seek to educate the whole child and overcome barriers to their learning, we all kind of have a piece and a part in that. And so, learning how to work together to address those needs is a big part of our job…Well, when you bring all the parties to the table. Um, you just have a broader understanding of various issues that are part of whatever challenge, you're facing. So I think you have a more comprehensive response when you when you work together and you have a whole system's approach versus just, you know, ‘well, this is what I think is wrong’ and that might just be a piece of the puzzle and not the entire picture. (School guidance counselor #2) |
| **Aim 3:** Lessons-learned and recommendations | |
| Inclusion of diverse voices | It assembles people from diverse backgrounds. Each of whom has some relevant piece of information to add that other people may not because of the diversity of their backgrounds. And I really feel like when you draw that type of insight together, from such a diverse group of people that it's a different but valid way of getting at the truth. And so I think it’s what I would recommend it to another community because I think it'd be good for the community...I would say that the program doesn't work if you don't have a lot of different segments of the community represented and if you're finding that most of the people are from one group, that it's probably not going to work all that well. You need people who can identify problems, people who can understand problems, who can effect change in the community. You just need a lot of different types of people with different types of expertise to come together. (Physician) |
| The role of leadership and organizers | I assume that without the right people, leading it like [names redacted], for example, that it could just become you know, a well intended event or activity that doesn't actually drive to the right leverage point. And so I just think it's important, how it's facilitated right. (Principal)  I also think that um with [ROI organizers] being who they are, and had anybody different come in to try to do what they are doing, I don't think the outcome would be the same. I think that their openness and willingness to understand and meet people where they are had a profound effect on how this is actually working out, you know, and it's no, no big Is and little Ts when it comes to them, we're all on the same playing field, which is what it should be. And it has made, it has allowed people who I work with closely, with ROI, I would have never thought that they would have done some of the things that they're doing, had it not been for [ROI organizers], or been in a place where they could even have the opportunity to do it. (School guidance counselor #1)  And I guess the whole process of how [ROI] have reached out to the entire community, you know, health services, professionals, school professionals. The clergy, faith-based organizations, just that broad reach that they've touched almost every aspect of the community, left no stone unturned in this work to raise awareness about ACEs and trauma and to provide the, the resources for resilience. (School guidance counselor #2) |
| Time and effort | Again feet on the ground hands to the handle.It's just going to take that sort of thing, it, it, it has taken generations to break and be broken, and it's going to take generations to fix. We got to have people who are willing to change, willing to fix things, willing to self disclose, willing to self examine, and, and so yes it'll take a lot of work in every community. (Higher education representative)  It is hard work. It does call you to…come, you know, come to grips with some things that even maybe you didn't know, and you may experience some things that you didn't expect to experience, but the end result is well worth it. (School guidance counselor #1)  The truth hurts. And this hurt, to see where it takes you. But if you, if you don't travel that journey. You don't feel that pain, you don't understand it and you're not gonna have any chance at all, any chance to rectify. And you got to also understand, this is not a quick fix. It's a generational investment in time and resources. I will be dead and gone, before, if we start on this program like we should and get the resources, before you really begin to see a meaningful improvement. But you know what? That's okay. Because I don't want my grandchildren, having the conversation that you and I are having right here. And I don't want those kids’ grandchildren that are growing up now, to have this, have to have that same conversation, or be in prison, because they were stealing to eat. (Local government employee #1)  Don't quit. Um, there's really something to this work and it's not a quick work. It's going to be a work that takes a lot of us, all of us working together and takes a commitment over time.  And a commitment that we see through because the need is never going to go away, there are always going to be new kids. There's always going to be trauma, there's always going to be someone that needs help. So just a commitment to continue the process. (School guidance counselor #2) |
