# Supplementary material for: Using systems-mapping to address Adverse Childhood Experiences (ACEs) and trauma: A qualitative study of stakeholder experiences
Source: PLoS One. 2022 Aug 18;17(8):e0273361. doi: 10.1371/journal.pone.0273361 (PMC9387783; doi:10.1371/journal.pone.0273361)
Supplement: Attachment — Submitted filename: PONE-D-22-02363 Response to Reviewers .docx [file pone.0273361.s004.docx]

June 23, 2022

Dear Dr.Wendel,

Thank you for the opportunity to submit a revised version of our manuscript titled “Using Systems-Mapping to Address Adverse Childhood Experiences (ACEs) and Trauma: A Qualitative Study of Stakeholder Experiences” (PONE-D-22-02363). We appreciate the time and effort you and the reviewers’ have taken to provide feedback. We have incorporated changes to reflect most of the edits suggested by the reviewers. Please note that line numbers listed are from the paper *without* tracked changes. Below are point-by-point responses to the reviewers’ comments:

**Comments from Reviewer 1**

1. **Comment:** *Describe what “1 ACES” vs “3 ACES” means. How are ACEs calculated?*

**Response:** We agree with this change. A clearer definition of how ACEs were calculated by the BRFSS has been included in lines 52-57.

2. **Comment:** *line 63 pg 3: change “attributable” to “associated with” & move the sentences about healthcare costs to the next paragraph after you talk about the kind of diseases/illnesses that ACEs contribute to*

**Response:** We agree with this change. “Attributable to” has been changed to “associated with” on line 70. The section about healthcare costs has been moved to lines 69-72 after the sentences about diseases.

3. **Comment:** *Elaborate on current individual level interventions and the current gap*

**Response:** We agree with this change. Lines 78-86 now highlight individual level interventions and the current gaps.

4. **Comment:** *Systems thinking is not a “critical component” of CBPR. CBPR can include systems thinking, but it can also be used to address individual level factors. I would argue that systems thinking, which is transdisciplinary in nature, requires community-based participation (in public health) but CBRP does not necessarily require systems thinking.*

**Response:** We agree with this suggestion. This phrase has been removed.

5. **Comment:** *The systems field typically refers to systems mapping in more specific techniques. In practice, it can be called system mapping. I suggest redefining systems mapping as community-based systems dynamics or causal loop diagramming because this paper is for a technical audience, not community partners.*

**Response:** We agree with this suggestion. Lines 94-97 now refer to a systems map as the more common term and causal loop diagramming as the specific technique.

6. **Comment:** *Define systems thinking and cite it and systems mapping.*

**Response:** We agree with this change. Lines 89-101 now provide definitions of systems thinking and systems-mapping with citations.

7. **Comment:** *I don’t know if I agree with the statement on lines 93-95. Do you have a citation?*

**Response:** A citation has been provided on line 115.

8. **Comment:** *The sentence on line 99-100 needs to be reworded and less specific. Describe the overall type of NP and rename this section “Systems Mapping Process” not “project”*

**Response:** We agree with this change. The sentence has been reworded on line 131 and “Project” has been changed to “Process” on line 130.

9. **Comment:** *Add more details to mapping process.*

**Response:** We agree with this change. Lines 142-176 includes detailed steps of the mapping process.

10. **Comment:** *Move section about participants to methods & more detail about the participants. Where did they come from? Incentives, recruitment strategy, response rate etc. Was 16 the total # of people recruited?*

**Response:** Thanks for this suggestion.

1) The authorship team considered the advantages and challenges of moving the participants section to methods and ultimately decided we prefer to leave it as is, following the examples of previously published qualitative papers in PLoS One where the participant information was included in the results section (Lasseter et al., 2018; Marcus-Varwijk et al., 2019; Nardi et al., 2020). If the editor feels strongly that we should reconsider our decision we are open to discussing it.
2) We have added more details about the participants in lines 243-245.
3) Incentives can be found on line 212. Sampling and recruitment information, and response rate (total reach of 200 individuals) are listed in lines 193-198.

11. **Comment:** *Describe study design (qualitative) first then talk about investigators. How many investigators?*

**Response:** We agree with this suggestion and have moved study design to the beginning of the Results section on lines 181-191. We have also clarified that the investigators include: graduate students TV, JB, LM, LV, and DE in line 203; ROI staff SS in line 202; and faculty SG in line 203.

12. **Comment:** *Were interview guides developed based on past literature? Why did you ask the questions you did? What was the overall research /evaluation question being addressed?*

**Response:** We agree with this suggestion. This information has been added to lines 182-185, and 203-208.

13. **Comment:** *I suggest using COREQ or SRSR guidelines for qualitative research to describe the methods. Look up the Equator Network to find the reporting guidelines.*

**Response:** We agree with this suggestion. We have reformatted the Methods section to address the COREQ guidelines and provided a citation on lines 190-191.

14. **Comment:** *How many coders?*

**Response:** There were 4 coders (line 220) working in two teams of 2 (line 226)

15. **Comment:** *What was inter-rater reliability? This is a statistic that is calculated.*

**Response:** We did not calculate an inter-rater reliability statistic because the coding team met to discuss each code application for each transcript. Thus, each transcript was verified for coding accuracy and agreement by at least 2 coders. Additionally, discrepancies were also brought to the entire research team (TV, JB, LM, LV, DE, SG, and SS) for group consensus. Our Methods reporting are in line with previously published PLoS One qualitative papers where the inter-rater reliability statistic was also not presented ((Lasseter et al., 2018; Marcus-Varwijk et al., 2019; Nardi et al., 2020).

16. **Comment:** *Based on your first finding, do you think that the SMP was an intervention within itself? It created a shared understanding and individual awareness based on your results, so it could have psychological benefits among others? I think this would be an interesting thing to add to the discussion.*

**Response:** We agree that this is an intriguing discussion point that should be added. An additional paragraph addressing this point has been added in lines 480-495.

17. **Comment:** *Discuss the actions resulting from SM in more detail. I think that this study is unique because it used SM to make changes. A lot of research uses SM to conceptualize issues, but doesn’t take the next steps.*

**Response:** We agree with this suggestion. Additional details about actions resulting from the SMP has been added in lines 462-270.

**Comments from Reviewer 2**

18. **Comment:** *The aim of this study needs to be much clearer at the end of the introduction. The lines 95-97 I think are the aims of this study but it would be helpful if they were framed more from the perspective of "the aim of this study was to...."*

**Response:** We agree with this suggestion. We have reframed the aims in lines 125-128 for clarity.

19. **Comment:** *The aim, questions and description of the overarching systems science work are not clear and the powerpoint referenced - reference 26 cannot be accessed to understand this study better. This description needs to much more clearly state what the overall aim of the systems science research was, as well as any sub-objectives/research questions.*

**Response:** We agree with this suggestion. We have added lines 111-176 to describe how the overarching systems-science work, the SMP, and the qualitative interviews relate to and build off of each other. The link to the PowerPoint reference has been fixed and is now accessible.

20. **Comment:** *The interaction between these two studies (the overall systems science SMP project), and this sub project of analysing the process and understanding the impact, needs to be better described. At the moment it doesn't seem to work well to have the description of the ROI systems mapping project at the end of the introduction (lines 98-124). The introduction should end with the aim of the project being described in this manuscript. The detailed description of the SMP project may be better in the methods section under a section around context.*

**Response:** We agree with this suggestion. In addition to the changes made in point #19, we have moved the description of the SMP into the Methods section labeled “Context: The ROI Systems-Mapping Process”

21. **Comment:** *Line 129 The wording around purpose should be much more including "The purpose of this study was to....."*

**Response:** After reorganizing and rewording the last paragraph of the introduction, we have removed this sentence.

22. **Comment:** *It would be helpful to have a summary list of the themes before starting straight into each one. E.g. The analysis resulted in three major themes including.....*

**Response:** We agree with this suggestion. An additional section has been added under Results describing a summary of the themes in lines 262-269.

23. **Comment:** *At the moment there is not much in the introduction or discussion about the use of systems thinking in ACES/child protection/Child maltreatment research. It would be helpful in the background to have more about how this (or similar approaches) has been used elsewhere in this subject matter. Then in the discussion how the results, strengths and limitations within this study relate to previous studies using this methodology.*

**Response:** We agree with this suggestion. We have added additional information in the introduction about the use of systems-thinking in ACEs research in lines 101-110, as well as relating our study to this previously published research in the discussion in lines 439-443.

24. **Comment:** *line 119 needs an 'of' in between 'development' and 'several'*

**Response:** This change has been made in line 171.

We look forward to hearing from you and the reviewers regarding our submission and respond to any further comments and suggestions.

Sincerely,

Thi Vu, MPH

Corresponding Author

Social and Behavioral Sciences Department

Yale School of Public Health
[thi.vu@yale.edu](mailto:thi.vu@yale.edu)
Tel: 704-957-0137
