# Supplementary material for: Using systems-mapping to address Adverse Childhood Experiences (ACEs) and trauma: A qualitative study of stakeholder experiences
Source: PLoS One. 2022 Aug 18;17(8):e0273361. doi: 10.1371/journal.pone.0273361 (PMC9387783; doi:10.1371/journal.pone.0273361)
Supplement: Attachment — Submitted filename: PONED2202363 Response to Reviewers July23_2022.docx [file pone.0273361.s005.docx]

July 21, 2022

Dear Dr.Wendel,

Thank you for the opportunity to submit a revised version of our manuscript titled “Using Systems-Mapping to Address Adverse Childhood Experiences (ACEs) and Trauma: A Qualitative Study of Stakeholder Experiences” (PONE-D-22-02363). We appreciate the reviewers’ feedback and have incorporated changes to reflect their suggested edits. Below are point-by-point responses to the reviewers’ comments:

**Comments from Reviewer 2**

1. **Comment:** *Line 157 “LLC” is this an acronym – if so expand.*

**Response:** We agree with this suggestion. We have removed the term “LLC” on line 157 because the sentence following “Engaging Inquiry” already describes what the organization is.

2. **Comment:** *Lines 182-183 “: 1) What were the 183 perceived individual, interpersonal, and societal level impacts the SMP?”

   Should there be an ‘of’ between ‘impacts’ and ‘the’*

**Response:** We apologize for the typo. We added an “of” on line 183.

3. **Comment:** *Names were in manuscript - was this supposed to be a double blind review. If so in future please anonymise your manuscript.*

**Response:** We were informed by the journal that all authors’ names must be listed in the title page.

4. **Comment:** *Not sure why original manuscript was submitted in the resubmission. It was confusing initially.*

**Response:** We removed all former manuscript versions from the submission portal and are only submitting the most recent revised versions in this round.

We look forward to hearing from you and the reviewers regarding our submission and respond to any further comments and suggestions.

Sincerely,

Thi Vu, MPH

Corresponding Author

Social and Behavioral Sciences Department

Yale School of Public Health
[thi.vu@yale.edu](mailto:thi.vu@yale.edu)
Tel: 704-957-0137
